# What is the patient-reported outcome, complication rate and conversion to total knee arthroplasty in patients with tibial plateau fractures caused by high-energy compared to low-energy mechanisms of injury?

**DOI:** 10.1007/s00068-025-02810-0

**Published:** 2025-04-03

**Authors:** Thijs P. Vaartjes, Tijmen W. Kraai, Eelke Bosma, Fabian J. van der Sluis, Joost G. ten Brinke, Reinier de Groot, Harm Hoekstra, Job N. Doornberg, Nick Assink, Frank F. A. IJpma

**Affiliations:** 1 Department of Trauma Surgery, University of Groningen, University Medical Center Groningen, HPC BA13, Hanzeplein 1, 9713 GZ Groningen, The Netherlands; 2 Department of Trauma Surgery, Martini Hospital, Groningen, The Netherlands; 3 Department of Trauma Surgery, Isala Hospitals, Zwolle, The Netherlands; 4 Department of Trauma Surgery, Gelre Hospitals, Apeldoorn, The Netherlands; 5 Department of Trauma Surgery, Medical Spectrum Twente, Enschede, The Netherlands; 6 Department of Traumatology, KU Leuven, University Hospitals Leuven Gasthuisberg Campus, Leuven, Belgium; 7 Department of Orthopedic Surgery, University Medical Center Groningen, Groningen, The Netherlands; 8 Department of Orthopedic Trauma Surgery, Flinders Medical Center, Adelaide, Australia; 9 3D Lab, University of Groningen, University Medical Center Groningen, Groningen, The Netherlands

**Keywords:** Tibial plateau fractures, High-energy trauma, Low-energy trauma, Trauma mechanism, Patient-reported functional outcome, KOOS

## Abstract

**Purpose:**

Despite varying impact of high- and low-energy traumas, research comparing patient and fracture characteristics as well as patient-reported functional outcomes following these trauma mechanisms is limited. From a patient, doctor, and legal perspective, assessing the association between trauma mechanism and clinical outcome is important for managing expectations.

**Methods:**

A multicenter cross-sectional study was performed including 1066 patients treated for a tibial plateau fracture between 2003 and 2019. Patients completed the Knee injury and Osteoarthritis Outcomes Score (KOOS) at a mean follow-up of 6 ± 4 years. Trauma mechanisms were classified according to ATLS guidelines. Independent- samples t-test and chi-square test were used to assess differences in patient and fracture characteristics after high- or low-energy trauma. Linear regression analyzed the relationship between trauma mechanisms and KOOS-scores. The Fisher’s exact assessed differences in complications and conversion to total knee arthroplasty (TKA).

**Results:**

High-energy trauma mostly occurred in younger males and low-energy trauma in older females. High-energy trauma caused more Schatzker IV-VI fractures, resulted in more initial fracture displacement and needed more often surgical treatment (81% versus 67%; p = 0.002). Linear regression showed that high-energy trauma was associated with lower KOOS-scores. Patients after high-energy trauma had more complications (e.g. revision surgery [8% versus 2%; p =  < 0.001], mal- or nonunion [8% versus 2%; p =  < 0.001]) and conversion to TKA (15% versus 10%; p = 0.144).

**Conclusion:**

Only 12% of patients with tibial plateau fractures sustained these injuries due to high-energy trauma, which predominantly involved younger males and resulted in more severe fractures. High-energy trauma resulted in worse patient-reported outcomes, more complications, and conversions to TKA.

**Level of evidence:**

Level III, prognostic study.

## Introduction

Tibial plateau fractures, caused by both high- and low-energy trauma mechanisms, account for approximately 1–2% of all fractures [1]. Fractures caused by high-energy trauma usually occur due to motorized vehicles accidents or falls from height, whereas low-energy tibial plateau fractures often occur from bicycle accidents or a fall from standing height [2–4]. High-energy traumas might cause more complex fractures, often combined with severe soft tissue injury impairing functional outcomes [3, 5, 6]. Low-energy trauma often result in relatively simple fractures, however in older aged patients, complex fracture patterns are also seen after low-energy trauma because of age-related osteoporotic bone structure [3, 7–9]. Despite differences in impact of these trauma mechanisms there is limited research available that compares the patient and fracture characteristics, as well as patient-reported functional outcome after high- or low-energy traumas.

From patient-, doctor-, and legal perspectives, trauma mechanism might be of importance for treatment and recovery expectations. In general, worse outcomes could be expected after high-energy trauma mechanisms, as substantial force striking the knee can cause significant damage to cartilage, bone and soft tissues in these types of injuries. However, study findings regarding outcome after high- or low-energy trauma are inconclusive. Some studies indicate that high-energy traumas are associated with good or satisfactory patient outcome [10, 11], while others suggest it results in worse patient-reported outcomes at follow-up [12, 13]. Most studies on tibial plateau fractures do not consider the trauma mechanism as a factor associated with outcome [14–17]. These studies report outcomes for the entire study population, without taking into account the amount of energy that caused the injury. We hypothesized that patients with high-energy trauma have different patient characteristics, fracture types and prognosis than those with low-energy trauma. To adequately inform patients during counseling regarding their prognosis after high- or low-energy trauma, it is important to assess if there are differences in patient and fracture characteristic and whether the trauma mechanism influences the clinical outcome.

We therefore asked: (1) What are the differences in patient and fracture characteristics in patients with tibial plateau fractures caused by high- or low-energy trauma mechanisms? (2) What are the differences in clinical outcomes (i.e. patient-reported outcome, complications and conversion to total knee arthroplasty) between patients with tibial plateau fractures caused by high- versus low-energy trauma mechanism?

## Method

### Study design and setting

A multicenter cross-sectional study was performed on all patients with tibial plateau fractures who were treated between January 2003 and December 2019 at the University Medical Center Groningen (The Netherlands), Isala hospitals (The Netherlands), Gelre hospitals (The Netherlands), Martini hospital (The Netherlands), Medical Spectrum Twente (The Netherlands) and University Hospitals Leuven (Belgium). These include four Level 1 trauma centers and two Level 2 trauma centers. The study procedure was approved in all participating centers by the institutional review board (research number: 201800411) and was performed in accordance with the relevant guidelines and regulations. This study is reported in accordance with the STROBE (Strengthening the Reporting of Observational Studies in Epidemiology) guideline [18].

### Participants

All patients of 18 years and older, treated for tibial plateau fractures, who had a diagnostic CT-scan, that were still alive and had at least 1 year of follow-up were considered eligible for inclusion. The exclusion criteria included, preexisting comorbidities of the injured leg, unknown trauma mechanism, patients with no knowledge of the Dutch language and patients with an unknown residential address.

### Patient-reported outcomes

All eligible patients were asked to complete the standardized Knee Injury and Osteoarthritis Outcome Score (KOOS) questionnaire [19]. In addition, patients were asked whether they underwent conversion to a total knee arthroplasty (TKA). KOOS is a validated questionnaire consisting of five subscales: symptoms, pain, activities of daily living (ADL), function in sports and recreation, and knee-related quality of life (QoL) [19]. A normalized score (100 indicating no symptoms and 0 indicating extreme symptoms) was calculated for each subscale. Patients who had conversion to TKA were assigned an average KOOS score that was reported in a previous cohort of patients with KOOS scores before conversion to TKA [20]. These scores represent the situation as it was before conversion to TKA. The assumed KOOS sub-scores were 52 for symptoms, 45 for pain, 55 for ADL, 16 for sport and 27 for QoL. The minimal clinically important difference of each KOOS subscale is 9 for symptoms, 12 for pain, 10 for ADL, 9 for sports, and 14 for QoL [21].

### Trauma mechanism

Patients were divided into two groups based on the energy of the trauma mechanism: high-energy (HET) and low-energy trauma (LET). The trauma mechanisms of all patients were reassessed through consensus by three observers (TPV, TWK, FFAIJ). The energy of the trauma mechanism was defined according to the criteria of the Advanced Trauma Life Support (ATLS) guidelines (appendix 1) [22]. Patients who met at least one of these criteria were classified as high-energy trauma.

### Data sources and measurements

Baseline characteristics, treatment (surgical or nonsurgical), trauma mechanism, injury severity scores (ISS), and associated injuries were retrieved from the patients’ electronic records. All knee radiographs and CT images were reassessed through consensus by two observers (TPV, FFAIJ) with experience in tibial plateau fracture management according to the Schatzker classification systems [23]. Gap and step-off measurements were performed on CT images to describe the fracture displacement. Gap was defined as separation of fracture fragments along the articular surface and step-off as separation of fracture fragments perpendicular to the articular surface [16]. The maximum gap and step-off were measured by going through axial, coronal and sagittal CT slices. The scores from each subscale of the KOOS questionnaire were calculated from the patient survey of the responding patients.

### Primary and secondary study outcomes

The primary study goal was to determine whether there were differences in patient and fracture characteristics between patients with a tibial plateau fracture after high- or low-energy trauma. The secondary goal was to assess whether there were differences in mid-term clinical outcomes after high- versus low-energy trauma in terms of patient-reported functional outcome, complications (e.g. reoperations, revision surgery) and conversion to TKA during follow-up.

### Statistical analysis

SPSS software (version 28, IBM Corp) was used for statistical analyses. Continuous variables are presented as the mean and standard deviation (SD) for normally distributed data and median and IQR for nonnormally distributed data. First, the study population was divided into two groups based on trauma mechanism (high- or low-energy). Descriptive statistics were used to describe the study population, and we used independent- samples t-test for continuous variables and chi-square test for noncontinuous variables to assess differences between the groups in terms patient and fracture characteristics. For sub-analysis, the study population after high- or low-energy trauma was divided in groups based on the patients age (≤ 50 and ≥ 51), because from the age of 50 the prevalence of osteoporosis is increasing [24, 25]. We used independent- samples t-test for continuous variables and chi-square test for noncontinuous variables to assess differences in fracture characteristics between young and older aged patients. Secondly, we used linear regression to analyze the relationship between the trauma mechanism and KOOS score. The model was adjusted for five potential confounders, including age, sex, BMI, smoking and diabetes. A Fisher’s exact test was used for the noncontinuous variables to assess differences in complications and conversion to TKA between patients after high- and low-energy trauma. Significance level was set at p < 0.05.

### Analysis of the nonparticipants

For the nonresponse analysis, we used an independent- samples t-test for continuous variables and a chi-square test for noncontinuous variables. The nonresponse analysis showed that responders were slightly older than nonresponders (53 ± 15 years versus 51 ± 17 years; p =  < 0.001). There was not much difference in sex between responders and nonresponders (female 68% [725 of 1066] versus female 58% [518 of 890]; p =  < 0.001). Due to data protection and privacy legislation as well as medical ethical reasons, knee radiographs and CT images of the nonresponders were only available in the initiating center and one affiliated center (n = 545). There was no difference in fracture classification between responders and nonresponders (Schatzker I [11% versus 12%], Schatzker II [31% versus 27%], Schatzker III [17% versus 18%], Schatzker IV [13% versus 16%], Schatzker V [8% versus 6%], Schatzker VI [20% versus 21%] p = 0.778).

## Results

**Fig. 1:**
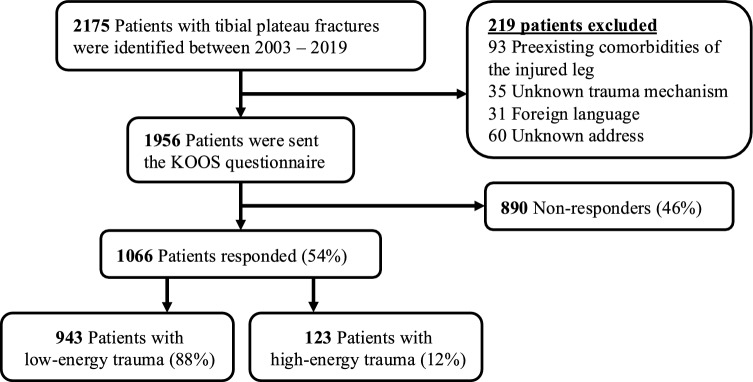
Patient inclusion and exclusion flow chart

A total of 2175 patients were treated for a tibial plateau fracture between 2003 and 2019. Of these patients, 93 had preexisting comorbidities of the injured leg, 35 had unknown trauma mechanism, 31 had no knowledge of the Dutch language and 60 had an unknown address, leaving 1956 patients for follow-up analysis. All eligible patients were contacted by posted mail from which 54% (1066 of 1956) responded at a mean follow-up of 6 ± 4 years. The mean age at the time of injury was 53 ± 15 years and 68% (725 out of 1066) of patients were female (Fig. 1).

### Difference in patient and fracture characteristics between high and low energy traumas

Between 2003 and 2019, 12% [123 out of 1066] of patients with tibial plateau fractures sustained their injuries due to high-energy trauma. High-energy traumas were mainly caused by road traffic accidents, whereas low-energy traumas were mainly caused by low energy falls and bicycle accidents (Table 1). High-energy trauma mostly occurred in younger males and low-energy trauma in older females (appendix 2). High-energy trauma caused more severe Schatzker IV-VI fractures (IV [27% versus 13%; p =  < 0.001], V [14% versus 6%; p = 0.002] and VI [24% versus 13%; p = 0.003]). Low-energy trauma caused more Schatzker II and III fractures (II [37% versus 23%; p = 0.002] and III [24% versus 10%; p =  < 0.001]). High-energy trauma resulted in more initial fracture displacement compared to low-energy trauma (gap [5.2 ± 5.5 versus 6.7 ± 5.7; p = 0.009], step-off [5.9 ± 5.9 versus 7.6 ± 7.0; p = 0.005]). Patients received more frequently surgical treatment after high-energy trauma compared to low-energy trauma (81% versus 67%; p = 0.002). High-energy trauma resulted in a higher prevalence of concomitant injuries (40% [49 of 123] versus 7% [62 of 943]; p =  < 0.001) (Table 1). The sub-analysis between young and older aged patients showed that low-energy trauma resulted in significantly more Schatzker VI fractures in the older aged patient population compared to the younger patients (16% versus 6%; p =  < 0.001) (Table 2).

**Table 1:** Differences in patient and fracture characteristics between high- and low- energy trauma

Patient characteristics	LET (n = 943)	HET (n = 123)	P-value
Age (years)	54 ± 15	48 ± 15	< 0.001
Female	682 (72)	43 (35)	< 0.001
BMI (kg/m^2^)	26.4 ± 4.7	26.6 ± 5.1	0.606
Smoking	193 (21)	19 (16)	0.167
Diabetic	78 (9)	7 (6)	0.295
Schatzker classification			
Schatzker I	71 (8)	4 (3)	0.091
Schatzker II	345 (37)	28 (23)	0.002
Schatzker III	231 (24)	12 (10)	< 0.001
Schatzker IV	119 (13)	33 (27)	< 0.001
Schatzker V	54 (6)	17 (14)	0.002
Schatzker VI	123 (13)	29 (24)	0.003
Initial fracture displacement			
Gap (mm)	5.2 ± 5.5	6.7 ± 5.7	0.009
Step-off (mm)	5.9 ± 5.9	7.6 ± 7.0	0.005
Treatment			
Surgical	627 (67)	99 (81)	0.002
Injury characteristics			
Left side	559 (59)	68 (55)	0.397
ISS			
> 16	0 (0)	14 (11)	< 0.001
Concomitant injuries			
Head	8 (1)	18 (15)	< 0.001
Chest	7 (1)	18 (15)	< 0.001
Abdomen	1 (0)	3 (3)	< 0.001
Pelvis	1 (0)	5 (4)	< 0.001
Upper limb	25 (3)	19 (15)	< 0.001
Lower limb	20 (2)	18 (15)	< 0.001
Trauma mechanism			< 0.001
Low- energy fall	456 (48)	0 (0)	
High- energy fall	0 (0)	4 (3)	
RTA**- pedestrian	26 (3)	11 (9)	
RTA- bicyclist	298 (32)	26 (21)	
RTA- scooter	42 (4)	24 (20)	
RTA- motorbike	14 (2)	33 (27)	
RTA- MVA	2 (0)	15 (12)	
Sports injury	73 (8)	5 (4)	
Other	32 (3)	5 (4)	
Follow-up (years)	6 ± 4	7 ± 4	0.257

*Numbers are presented as mean ± standard deviation or as number and percentage (%)

**RTA is road traffic accident

**Table 2:** Fracture characteristics of the young and older aged patients

Fracture characteristics	LET (n = 943)		P-value	HET (n = 123)		P-value
Age group	≤ 50 (n = 300)	≥ 51 (n = 643)		≤ 50 (n = 65)	≥ 51 (n = 58)	
Schatzker classification						
Schatzker I	37 (12)	34 (5)	< 0.001	2 (3)	2 (3)	0.908
Schatzker II	107 (36)	238 (37)	0.689	12 (18)	16 (28)	0.283
Schatzker III	72 (24)	159 (25)	0.809	7 (11)	5 (9)	0.689
Schatzker IV	51 (17)	68 (11)	0.006	17 (26)	16 (28)	0.858
Schatzker V	16 (5)	38 (6)	0.723	9 (14)	8 (14)	0.993
Schatzker VI	17 (6)	106 (16)	< 0.001	18 (28)	11 (19)	0.255

*Numbers are presented as mean ± standard deviation or as number and percentage (%)

### Differences in clinical outcome after high or low-energy trauma

Clinical outcome is subdivided in patient-reported outcome, complications (e.g. reoperations or revision surgery), and conversion to total knee arthroplasty. First of all, tibial plateau fractures resulted in worse patient-reported functional outcome after high-energy trauma. KOOS scores after high-energy or low-energy trauma differ in symptoms (71 ± 21 versus 76 ± 21; p = 0.008), pain (72 ± 23 versus 77 ± 22; p = 0.044) and QoL (51 ± 28 versus 59 ± 28; p = 0.005) except for sport (41 ± 34 versus 48 ± 34; p = 0.051) and ADL (76 ± 20 versus 79 ± 21; p = 0.068). After correction for potential confounders including age, sex, BMI, smoking and diabetes, linear regression showed that high-energy trauma mechanism was associated with lower KOOS scores for each of the five subscales of the KOOS questionnaire (Table 3). Secondly, patients after high-energy trauma had more reoperations, revision surgeries, and mal- or nonunions of the tibial plateau. Eventually, there was a slight difference in conversion to TKA after high- compared to low-energy trauma (15% versus 10%; p = 0.144) (Table 4).

**Table 3:** High-energy trauma associated with lower patient-reported outcome, a multivariate analysis

KOOS subscale	β^a^	95% CI	P-value
Symptoms	− 7.1	− 11.1 to − 3.0	< 0.001
Pain	− 7.0	− 11.3 to − 2.6	< 0.001
ADL	− 6.7	− 10.7 to − 2.7	< 0.001
Sport	− 11.7	− 18.5 to − 4.9	< 0.001
QoL	− 9.5	− 15.0 to − 4.0	< 0.001

^a^Adjusted for potential confounders including age, sex, BMI, smoking and diabetes

**Table 4:** High-energy trauma associated with more reoperations and complications

Reoperations and Complications	Total	LET	HET	P-value
Removal of osteosynthesis material	327 (31)	270 (29)	57 (46)	< 0.001
Reoperation for fracture-related infection	32 (3)	28 (3)	4 (3)	0.780
Reoperation for meniscal or ligamentous repair	30 (3)	23 (2)	7 (6)	0.072
Revision surgery for residual replacement	25 (2)	15 (2)	10 (8)	< 0.001
Malunion or nonunion	29 (3)	19 (2)	10 (8)	< 0.001
Conversion to Total Knee Arthroplasty	115 (11)	97 (10)	18 (15)	0.163

*Numbers are presented as number and percentage (%)

## Discussion

Despite the different impact of high- and low-energy trauma mechanisms, there is limited research available which compares the difference in patient characteristic, fracture types, and clinical outcome associated with these different levels of energy causing these injuries. To adequately counsel patients regarding their prognosis following high- or low-energy trauma, it is important to understand whether the type of injury influences the outcome. We evaluated a large cohort of patients with tibial plateau fractures after both high- and low-energy trauma to assess whether there are differences between these groups. First of all, our results showed that high-energy traumas mostly occurred in younger males and caused more severe fractures according to the Schatzker classification whereas low-energy trauma mostly occurred in older females [23]. Furthermore, Schatzker VI fractures occurred in 1 out of 6 older aged patients after low-energy trauma, compared to 1 out of 17 in younger patients. This indicates that older patients, who may have more osteoporotic bone, tend to experience more severe fracture patterns despite low-energy trauma. Secondly, our study demonstrated that the clinical outcome in terms of patient-reported functional outcome is slightly worse after high-energy trauma, but within the range of the minimal clinically important difference. The complication rate, however, is higher after high-energy trauma. The conversion rate to TKA is slightly higher after high-energy compared to low energy trauma (15% versus 10%). Based on these findings, we believe that patients, surgeons, and insurance companies should be aware that high-energy tibial plateau fractures account for only ± 12% of all tibial plateau fractures, but should be considered challenging injuries with worse outcome.

### Differences in patient and fracture characteristics after high- versus low-energy tibial plateau fractures

Tibial plateau fractures result from various high- and low-energy trauma mechanisms. Our study showed that high-energy traumas were mostly caused due to road traffic accidents (e.g. motorbike, scooter and bicyclist) and low-energy traumas due to low energy falls (e.g. fall from standing height) and bicycle accidents. High-energy traumas occurred more often in younger males and low-energy traumas in older females. Similar results are shown in a population-based study of Elsoe et al. who also reported that high-energy trauma occurred more often in younger patients and low-energy trauma in older females [3]. However, they describe an equally distribution of males and females with high-energy traumas in the younger aged population, while our data indicated these cases were predominantly males. Furthermore, our results showed that high-energy trauma more often caused severe Schatzker IV-VI fracture patterns and low-energy trauma mostly Schatzker II & III fractures. This corresponds to literature on tibial plateau fractures, which often classifies Schatzker I-III as low-energy fractures and more complex Schatzker IV-VI as high-energy fractures [2, 5, 6, 9]. Moreover, our study revealed that low-energy trauma caused more severe fracture patterns in older aged patients than in younger patients. Severe Schatzker VI fractures occurred in 16% of older aged patients with low-energy traumas, compared to 6% in younger patients. This finding is in line with other recent studies describing complex fracture patterns in older aged patients after low-energy trauma mechanism due to age related changes in bone structure (e.g. osteoporosis and osteoarthritis) [4, 7, 9]. Overall, high-energy traumas occur mostly in young males and result in more severe fracture patterns, whereas low-energy traumas are more common in older females but can result in severe fracture patterns as well, probably due to age-related decrease in bone density.

### Differences in clinical outcome after high- versus low-energy tibial plateau fractures

Research regarding differences in clinical outcome of patients with tibial plateau fractures after high- or low-energy trauma is limited. We found that high-energy trauma mechanisms resulted in worse patient-reported functional outcome (KOOS). However, the difference in scores remained within the range of the minimum clinically important difference except for sport (*B* of −11.7 [95% CI: −18.5 – −4.9]; p =  < 0.001) [21]. This suggests that while high-energy trauma leads to poorer functional outcomes, the impact on patient-reported outcomes is not beyond the threshold considered clinically significant. Furthermore, our study showed more complications (e.g. reoperations and revision surgery) after high-energy traumas, due to more complex fractures and extensive soft tissue damage. There was no substantial difference in rate of conversion to TKA between high- and low-energy trauma, although the percentage of TKA following high-energy trauma was slightly higher (15% versus 10%). To put this into perspective, the primary indication for TKA is elderly patients with degenerative or post-traumatic osteoarthropathy. In general, surgeons are more conservative in recommending TKA for younger patients due to the limited lifespan of prostheses caused by polyethylene wear. Therefore, a higher rate of conversion to TKA after high-energy trauma, primarily affecting younger patients, highlights the severity of such injuries and their worse outcomes compared to low-energy traumas. Literature comparing clinical outcome after high- versus low-energy trauma in tibial plateau fracture management is scarce. Timmer et al. assessed the functional outcome after tibial plateau fracture osteosynthesis in a small retrospective cohort of 82 patients [12]. Their study presented some preliminary results that high-energy trauma patients had worse patient-reported outcomes (KOOS pain and quality of life). However, their study had a limited small sample size and did not include patients who underwent conversion to TKA in the analyses between high- and low-energy trauma patients. The study of Assink et al. reported similar results regarding complications during follow-up and showed that 14% had conversion to TKA [26]. However, this studies only assessed surgically treated patients and did not distinguish between high- and low-energy trauma when reporting complications and conversion to TKA. The risk of conversion to TKA in the current literature range from 0 to 21% [27], which may be due to differences in study populations (e.g. case mix and variations in indications for TKA). It is therefore difficult to directly compare our results with other studies. However, a recent study demonstrated that increase of fracture displacement, as measured in 3D, is associated with conversion to TKA [14]. This means that severe fracture patterns, more often seen in relatively younger individuals following high-energy trauma, eventually have higher risks of conversion to TKA as they age. When treating patients after high-energy trauma it is important to understand that this could potentially result in worse patient-reported functional outcomes and more complications (e.g. reoperations and revision surgery).

### Limitations

This study has some response bias, which is inherent to the cross-sectional study design caused by loss of follow-up and nonresponse [28]. By approaching all eligible patients’ multiple times, we attempted to reduce this risk as much as possible. The response rate of 54% is relatively high considering the mean follow-up of 6 years and is in line with what could be expected according to literature on postal survey studies [29]. On the other hand, this is one of the largest multicenter studies comparing patient characteristics, fracture types, and clinical outcome after both high- and low-energy trauma, including over 1000 patients with tibial plateau fractures. The nonresponse analysis showed only minor differences in terms of age, sex and fracture classification between the responders and nonresponders, which is unlikely to affect our findings. Another limitation was the high variation in follow-up time, which ranged from 1 to 17 years (mean 6 ± 4) and is inherent to the cross-sectional study design. Therefore, our findings regarding clinical outcome between patients after high- or low-energy trauma mechanism are applicable for midterm follow-up but should be interpreted with caution regarding long-term follow-up (20 + years). Furthermore, the quality of fracture reduction was not considered when interpreting patient-reported outcome scores and the complication rate during follow-up. Lastly, high-energy trauma caused more concomitant injuries, which is according clinical practice but could have influenced the patient-reported functional outcomes.

## Conclusion

Only 12% of tibial plateau fractures were caused by high-energy traumas, typically affecting younger males in road traffic accidents. Low-energy traumas mostly occurred in older females from low energy falls and bicycle accidents. High-energy trauma resulted in more severe fracture patterns, although similar fracture patterns were seen in older patients after low-energy trauma, likely due to age-related decrease in bone density. High-energy trauma resulted in slightly worse patient-reported functional outcome, although these differences did not exceed the minimal clinically important difference except for sport. Furthermore, it resulted in more complications due to the complex fractures and extensive soft tissue injury. There was no substantial difference in conversion to TKA between patients after high- or low-energy trauma. Physicians, surgeons, rehabilitation doctors, and even legal personnel should be aware that management of patients with tibial plateau fractures after high-energy trauma is challenging, may require some dedicated teams and could result in worse clinical outcome. Future, large prospective studies are needed for personalized risk stratification and patients counselling after high- or low-energy trauma to further facilitate the shared-decision making process.

## Figures and Tables

**Fig. 2:**
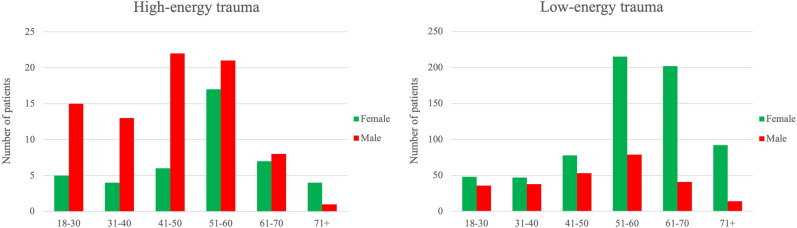
Age distribution of patients with tibial plateau fractures after high-energy trauma and low-energy trauma

## Data Availability

No datasets were generated or analysed during the current study.

## Appendix

See Table 5.

**Table 5:** ATLS criteria for high-energy trauma mechanisms

Falls Adults > 20 feet of 6 m (2 stories) Childeren > 10 feet or 3 m (2–3 × height of child)
High-risk motor vehicle crash Intrusion, including roof: > 12 inches (30 cm) occupant side, > 18 inches (45 cm) any side Ejection (partial or complete) from vehicle Death in same passenger compartment Vehicle telemetry data consistent with high risk of injury
Auto versus pedestrian/byciclist Thrown, run over, or with significant (> 20 mph; 32 kph) impact
Motorcycle crash > 20 mph

See Fig. 2.
